# Lightweight Hash-Based Authentication Protocol for Smart Grids

**DOI:** 10.3390/s24103085

**Published:** 2024-05-13

**Authors:** Sangjin Kook, Keunok Kim, Jihyeon Ryu, Youngsook Lee, Dongho Won

**Affiliations:** 1 Department of Electrical and Computer Engineering, Sungkyunkwan University, 2066 Seobu-ro, Jangan-gu, Suwon-si 16419, Republic of Korea; sangjinkook@gmail.com (S.K.); kimkeunok@gmail.com (K.K.); 2 School of Computer and Information Engineering, Kwangwoon University, Seoul-si 01897, Republic of Korea; jhryu@kw.ac.kr; 3 Department of Computer Information Security, Howon University, 64 Impi-myeon, Howondae 3-gil, Gunsan-si 54058, Republic of Korea; ysooklee@howon.ac.kr

**Keywords:** smart grid authentication, lightweight user authentication, hash-based authentication

## Abstract

Smart grids integrate information and communications technology into the processes of electricity production, transportation, and consumption, thereby enabling interactions between power suppliers and consumers to increase the efficiency of the power grid. To achieve this, smart meters (SMs) are installed in households or buildings to measure electricity usage and allow power suppliers or consumers to monitor and manage it in real time. However, SMs require a secure service to address malicious attacks during memory protection and communication processes and a lightweight communication protocol suitable for devices with computational and communication constraints. This paper proposes an authentication protocol based on a one-way hash function to address these issues. This protocol includes message authentication functions to address message tampering and uses a changing encryption key for secure communication during each transmission. The security and performance analysis of this protocol shows that it can address existing attacks and provides 105,281.67% better computational efficiency than previous methods.

## 1. Introduction

A smart grid (SG) is an advanced power-grid system that integrates information and communications technologies to enhance the efficiency and reliability of electricity production, transportation, and consumption [1]. These systems enable intelligent demand management, the linkage of new and renewable energies, and electric vehicle charging through real-time information exchange between suppliers and consumers [2]. As the sales of electric vehicles and power consumption increase significantly every year, SGs and related security issues have become more important [3]. One of the key components of the SG is the deployment of smart meters (SMs) in households and buildings [4,5,6,7,8,9,10], which enable the real-time monitoring and management of electricity usage by both power suppliers and consumers.

Information monitored in real time is important for security [11]. For example, if electricity usage is leaked outside, an attacker can determine whether a house is empty, and by analyzing this information, they can also determine the living patterns of the individual. This is an important personal privacy issue, as individuals may become involved in crimes or undesirable events against their will. In another example, problems may occur if electricity usage is falsified. Attackers may attempt to make financial gains by reducing their own usage; conversely, attackers may increase their usage and cause inconvenience to neighbors with whom they do not get along.

However, the security of SMs and their communication protocols is of paramount importance for preventing malicious attacks and ensuring the integrity and confidentiality of data. To address these security concerns, this paper introduces a hash-based lightweight authentication scheme specifically designed for SG environments. The proposed authentication scheme aims to provide a secure and efficient method for authenticating communication between SMs and power suppliers while considering the computational and communication constraints of these devices.

The primary objective of the authentication scheme is to ensure the following:

- *Secure memory protection:* The scheme addresses the need for secure memory protection in SMs to safeguard against the unauthorized access and tampering of sensitive data stored within the devices.
- *Robust communication security:* By employing a lightweight communication protocol, the scheme ensures secure communication between SMs and power suppliers, protecting against eavesdropping, message tampering, and replay attacks.
- *Efficient computational requirements:* Recognizing the resource limitations of SMs, the proposed scheme aims to minimize the computational overhead, ensuring efficient authentication without compromising security.

Recently, researchers [4,5,6,7,8,9,10] have conducted studies on the security of SMs and their communication protocols; however, several of these studies [4,5,6,7,8,9,10] have failed to satisfy the various security requirements outlined earlier. In 2021, Aghapour et al. [10] published a study on lightweight cryptography. However, our study demonstrates that Aghapour et al. [10]’s study has vulnerabilities, such as inferred data reports, extracted keys, and the potential for message recovery. Therefore, a new authentication protocol is required for SGs.

We propose a scheme that satisfies these requirements. Our scheme is designed to provide secure memory protection and has been verified to satisfy ten security requirements, ensuring robust communication security. Our scheme is based on a one-way hash function and utilizes message authentication functions and changing encryption keys to satisfy efficient computational requirements. Through a comprehensive security and performance analysis, the proposed scheme demonstrates its effectiveness in addressing existing attacks and achieving better computational efficiency than previous studies.

The remainder of this paper is organized as follows: In Section 3, we present the hash functions of the system and attack models. The target scheme is introduced in Section 4. Section 5 describes the limitations of the proposed scheme. The proposed scheme is presented in Section 6. In Section 7, we provide formal and informal security analyses. In Section 8, we present a performance analysis of the proposed scheme, and in Section 9, we discuss the results. Finally, we conclude this paper in Section 10.

## 2. Related Work

In the field of SG security, several studies have proposed lightweight authentication schemes that address the unique challenges and requirements of SG environments.

In 2018, Mahomood et al. [4] proposed an authentication scheme based on elliptic curve cryptography (ECC) to satisfy the complex security requirements of SGs. In 2021, Sadhukhan et al. [6] introduced an ECC-based SG communication authentication scheme comprising a trusted authority, an SM, and a service provider. Sadhukhan et al. [6]’s scheme defends against impersonation attacks, which Mahomood et al. [4]’s scheme fails to protect against, and additionally satisfies, SM anonymity and data confidentiality. In 2021, Sureshkumar et al. [7] designed a scheme for the communication between service providers and SMs. However, Sureshkumar’s method is vulnerable because it does not use a one-time pad key. Furthermore, in 2023, Hu et al. [5] pointed out that Mahomood et al. [4]’s scheme does not ensure user anonymity and is vulnerable to ephemeral secret leakage attacks, and hence proposed an authentication and key agreement scheme for SGs with enhanced security based on ECC.

Recently, several authentication schemes for SG environments that do not use ECC have been proposed. In 2020, Kaveh and Mosavi [8] introduced an authentication scheme for SG environments using a physically unclonable function to counteract attacks involving physical replication or damage. Recently, Tanveer and Alasmary [9] proposed an authentication scheme for SG environments using the new hash function “Esch256”. In 2021, Aghapour et al. [10] proposed a fully lightweight two-way communication scheme for SG environments. Aghapour et al. [10] utilized only one-way hash functions and XOR operations for authentication between the participants, making their scheme the most lightweight one. However, in this study, we identified a critical vulnerability in Aghapour et al. [10]’s scheme. Their scheme enables the extraction of keys when data reports are inferred, and messages can be recovered based on the extracted key.

## 3. Preliminaries

In this section, the hash function, system model, and attack model are described. The details are as follows:

### 3.1. Hash Function

In this study, we adopt a hash function as an algorithm for verifying messages or for generating keys [12,13,14]. Hash functions are widely known to have the following four main characteristics:

- *Compute a hash function efficiently:* The calculation of the hash value by the hash function must be fast, regardless of the size of the input data.
- *Preimage resistance:* For the hash function h(·), given y=h(x), it should be computationally infeasible to find *x*.
- *Second preimage resistance:* For the hash function h(·), given *x*, it should be computationally infeasible to find another $x_{2}\neq x$ such that $h\left(x\right)=h\left(x_{2}\right)$.
- *Collision resistance:* For the hash function h(·), it should be computationally infeasible to find $x_{1}$ and $x_{2}$, where $x_{1}\neq x_{2}$ such that $h\left(x_{1}\right)=h\left(x_{2}\right)$.

Furthermore, recent studies have shown that widely used hash functions, such as MD4, MD5, SHA1, RIPEMD-160, SHA2-256, and SHA-512, are prone to issues, such as collision resistance, second preimage resistance, and no length extension, owing to advances in computational speed [15]. Therefore, we assume that the hash function used in our scheme is the most recently developed and has yet to be found to be vulnerable: SHA3-256.

### 3.2. System Model

**Figure 1:**
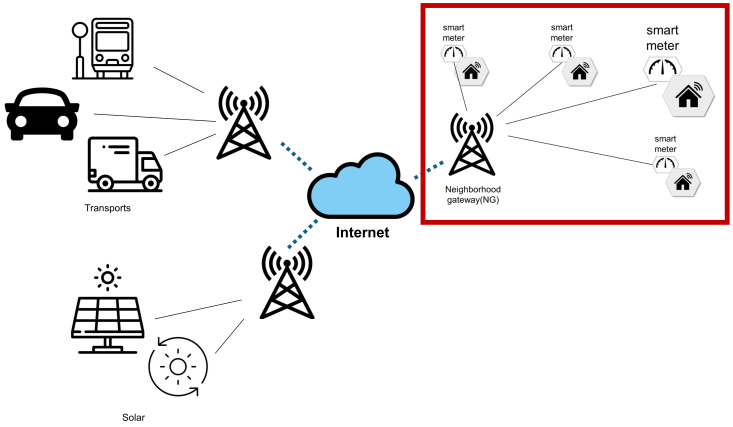
A system model where the smart meter and neighborhood gateway communicate with other neighborhoods’ edge nodes over the internet.

We proposed a scheme for communication between SMs and power supplier servers in an SG environment [16,17]. The two nodes that participate in the communication possess a hierarchical communication model as illustrated in Figure 1.

Smart grids provide bidirectional services; thus, automated communication occurs over public channels. If certain nodes provide incorrect status and situational information, the microgrid controlled by these nodes is at risk of being compromised [18]. Furthermore, while current smart grids are easily deployable and modifiable, they must be carefully designed due to the various existing cyber threats they face [19].

Smart grids have long been subject to attacks worldwide. In 2009, a senior analyst at the US CID reported that Russian and Chinese cyber spies had penetrated the US power grid [20]. In December 2016, Russia attacked Ukraine’s energy grid, which resulted in opening the circuit breakers of Ukraine’s energy grid and caused a power outage for about an hour [21].

Attacks on smart grids typically originate from the information sent from endpoint devices to common nodes such as neighborhood gateways. Attackers who infiltrate the smart grid network through these devices can then exploit vulnerabilities in the central control system to take over the smart grid. Subsequently, attackers may attempt attacks such as power shutdowns and personal data breaches through the control system, causing damage. To defend against such attacks, the FERC uses emergency orders and sanctions related to the cyber security of the power infrastructure [22], while NIST sets standards to ensure all systems in the smart grid are interoperable [23].

The details regarding the participating smart meters (SMs) and neighborhood gateways (NG) are as follows:

- *Smart meter (SM):* An electronic device that measures the consumption of utilities, such as electricity, gas, and water, collecting data in real time. It communicates with the neighborhood gateway to transmit data reports. Users utilize SMs to monitor their energy usage.
- *Neighborhood gateway (NG):* A neighborhood gateway is configured within a neighborhood area network and communicates regularly with dozens to hundreds of smart meters. For example, it could be installed in a commercial building’s technical room, where it serves the role of transmitting data to a central energy management system, or it might be placed within a home to monitor the household’s energy consumption. In the case of a residential gateway, it could be connected via Bluetooth, Zigbee, or Wi-Fi, and typically supports a capacity of 128 MB or more [24,25]. At a minimum, the gateway must store the information from the smart meter until it can be sent to the cloud or the company. The neighborhood gateway enables smart meters to exchange information with the cloud or the company. It requests data from each SM and collects their data. The neighborhood gateway checks the confidentiality and integrity of the data collected from the SMs.

### 3.3. Attack Model

We propose a scheme based on the threat model suggested by Dolev–Yao [26,27]. The main characteristics of the Dolev–Yao model [26] are as follows:

- The attacker eavesdrops on all the transmission packets used in the public channel.
- The attacker attempts to decrypt the eavesdropped transmission packets to obtain the values (data report, message, etc.) intended for transmission through communication.
- The attacker attempts to alter the messages used in communication by performing a man-in-the-middle attack.
- The attacker attempts a replay attack.

In this paper, we propose a scheme that defends against these attacks and demonstrate its resistance to them.

## 4. Review of Aghapour et al.’s Scheme [10]

In this section, we introduce the target scheme suggested by Aghapour et al. [10]. Their scheme consists of an initialization phase and a secure communication phase.

### 4.1. Initialization Phase

In Aghapour et al. [10]’s scheme, at this stage, each *j*-th $SM_{j}$ registers its identity $ID_{j}$ with a neighborhood gateway (NG). NG then transmits an initial secret key value $K_{0}^{j}$ to each SM over a secure channel. Subsequently, NG stores the pair of the SM identity and secret key ($ID_{j}$, $K_{0}^{j}$) in its database, and each SM $SM_{j}$ stores the initial secret key value $K_{j}^{0}$ in its memory.

### 4.2. Secure Communication Phase

In the stage proposed by Aghapour et al. [10], message authentication between the *j*-th SM $SM_{j}$ and NG occurs over a public channel. The details are as follows.

#### 4.2.1. First Authentication

1. NG generates the random number $r_{i}^{j}$ for $SM_{j}$. NG computes $A_{i}^{j}=\left(\left(m_{i}^{j}⊕r_{i}^{j}\right)\|r_{i}^{j}\right)⊕K_{i}^{j}$, $V_{i}^{j}=H(m_{i}^{j}\|r_{i}^{j}\|ID_{j}\|T_{NG}\left\|K_{i}^{j}\right)$, where $m_{i}^{j}$ is the *i*-th message for $SM_{j}$, $T_{NG}$ is a timestamp of NG, and H(·) is a one-way hash function. NG sends a message $M_{1}=\{A_{i}^{j}$, $V_{i}^{j}$, $T_{NG}$, $ID_{j}\}$ to $SM_{j}$ in the public channel.
2. $SM_{j}$ receives the message $M_{1}=\{A_{i}^{j}$, $V_{i}^{j}$, $T_{NG}$, $ID_{j}\}$ from NG, and computes $\left(m_{i}^{j}⊕r_{i}^{j}\right)\|r_{i}^{j}=A_{i}^{j}⊕K_{i}^{j}$ to obtain $r_{i}^{j}$ and $m_{i}^{j}$. $SM_{j}$ verifies $V_{i}^{j}=h(m_{i}^{j}\|r_{i}^{j}\|ID_{j}\|T_{NG}\left\|K_{i}^{j}\right)$. If it fails to verify the message, $SM_{j}$ stops the protocol. If its verification succeeds, the authenticity of NG is verified by $SM_{j}$, and the first authentication phase ends.

#### 4.2.2. Second Authentication

1. $SM_{j}$ computes $E_{i}^{j}=\left(h\left(r_{i}^{j}\right)\|D_{i}^{j}\right)⊕K_{i}^{j}$, where $D_{i}^{j}$ is the data report from the corresponding SM, and h(·) is a different hash function with H(·). $SM_{j}$ creates the new key $K_{i+1}^{j}=H(r_{i}^{j}\|ID_{j}\|T_{j}\left\|K_{i}^{j}\right)$, where $T_{j}$ is a timestamp of $SM_{j}$. It replaces the old key $K_{i}^{j}$ with $K_{i+1}^{j}$. $SM_{j}$ makes the verification $V_{i}^{\prime j}=H(D_{i}^{j}\|r_{i}^{j}\|ID_{j}\|T_{j}\left\|K_{i+1}^{j}\right)$ and sends a message $M_{2}=\{E_{i}^{j}$, $V_{i}^{\prime j}$, $T_{j}\}$ to NG.
2. NG receives the message $M_{2}=\{E_{i}^{j}$, $V_{i}^{\prime j}$, $T_{j}\}$ from $SM_{j}$ and computes $\left(h\left(r_{i}^{j}\right)\|D_{i}^{j}\right)=E_{i}^{j}⊕K_{i}^{j}$. NG computes $K_{i+1}^{j}=H(r_{i}^{j}\|ID_{j}\|T_{j}\left\|K_{i}^{j}\right)$. NG verifies $V_{i}^{\prime j}=H(D_{i}^{j}\|r_{i}^{j}\|ID_{j}\|T_{j}\left\|K_{i+1}^{j}\right)$, and if its verification succeeds, NG compares $D_{i}^{j}$ with the existing format and stores $K_{i+1}^{j}$ in its database.

## 5. Limitations of Aghapour et al.’s Scheme [10]

We identified a critical vulnerability in the scheme proposed by Aghapour et al. [10] as previously described. In this section, we discuss the vulnerabilities identified in Aghapour et al. [10]’s scheme. The details are as follows:

### 5.1. Inferrability of the Data Report

We assume that the data report $D_{i}^{j}$ can be inferred because it has a similar format. This is likely because the data report $D_{i}^{j}$, such as electricity usage, tends to be within a certain range of the actual values.

### 5.2. Inferrability of the Message

We can obtain the values of $A_{i}^{j}$ and $E_{i}^{j}$ using the values in $M_{1}$ and $M_{2}$ transmitted over the public channel. Using the obtained $A_{i}^{j}$ and $E_{i}^{j}$ values, we derive the following equation:

(1) $$A_{i}^{j}⊕E_{i}^{j}$$

(2) $$=\left(\left(\left(m_{i}^{j}⊕r_{i}^{j}\right)\|r_{i}^{j}\right)⊕K_{i}^{j}\right)⊕\left(\left(h\left(r_{i}^{j}\right)\|D_{i}^{j}\right)⊕K_{i}^{j}\right)$$

(3) $$=\left(\left(m_{i}^{j}⊕r_{i}^{j}\right)\|r_{i}^{j}\right)⊕\left(h\left(r_{i}^{j}\right)\|D_{i}^{j}\right)$$

Here, we assume that we can estimate $D_{i}^{j}$ according to Section 5.1; thus, we obtain the value of $r_{i}^{j}$. In addition, we obtain $h(r_{i}^{j})$ using $r_{i}^{j}$. Finally, we can derive the message $m_{i}^{j}$ using the previously obtained $r_{i}^{j}$, $h(r_{i}^{j})$, and $D_{i}^{j}$.

### 5.3. Extraction of the Secret Key

In Section 5.2, we obtained $r_{i}^{j}$, $m_{i}^{j}$, and $D_{i}^{j}$. Using these variables, we derived the secret key value $K_{i}^{j}$ using $A_{i}^{j}$. This is derived as follows:

(4) $$A_{i}^{j}=\left(\left(m_{i}^{j}⊕r_{i}^{j}\right)\|r_{i}^{j}\right)⊕K_{i}^{j}$$

(5) $$K_{i}^{j}=\left(\left(m_{i}^{j}⊕r_{i}^{j}\right)\|r_{i}^{j}\right)⊕A_{i}^{j}$$

## 6. Proposed Scheme

In this section, we propose enhanced hash-based authentication in SGs to address the vulnerabilities identified in Section 5. The notations used in this paper are explained in Table 1. The details are as follows:

### 6.1. Initialization Phase

**Figure 2:**
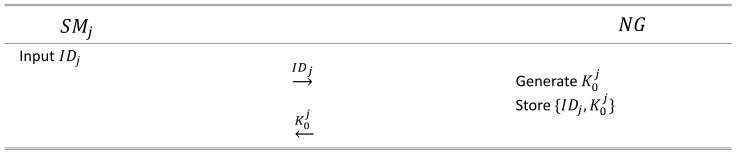
The phase of registering the identity $ID_{j}$ of the smart meter $SM_{j}$ with the neighborhood gateway NG proposed in this study.

In this phase, NG verifies the identity of each SM and assigns an initial secret key individually. The details are shown in Figure 2.

1. We denote the *j*-th SM as $SM_{j}$. At this time, $SM_{j}$ selects its own identity information. When the identity chosen by $SM_{j}$ is denoted as $ID_{j}$, $SM_{j}$ transmits the $ID_{j}$ information to NG through a secure channel.
2. NG receives the identity information of each SM through a secure channel. Assuming that it receives the identity $ID_{j}$ of the *j*-th SM, NG generates an initial secret key $K_{0}^{j}$ for communication with $SM_{j}$. NG then stores the pair $ID_{j}$, $K_{0}^{j}$ in its database. NG transmits the generated $K_{0}^{j}$ to $SM_{j}$ through a secret channel, and $SM_{j}$ receives and stores the secret key $K_{0}^{j}$.

### 6.2. First Secure Communication Phase

**Figure 3:**
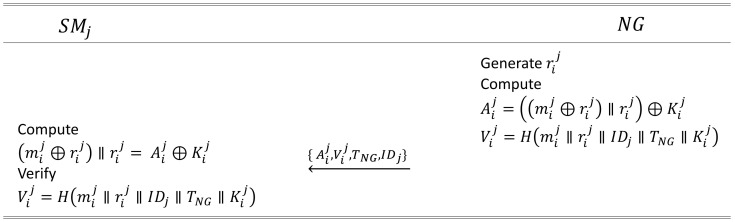
The first authentication phase between smart meter $SM_{j}$ and neighborhood gateway NG proposed in this study.

In this phase, NG sends information to the *j*-th SM $SM_{j}$ through a public channel, protecting it from external leakage using hashing and concatenation operations. $SM_{j}$ checks the message received from NG and verifies its integrity. The details are presented in Figure 3.

1. To securely send a message to $SM_{j}$, NG generates a random number $r_{i}^{j}$ and a timestamp $T_{NG}$. To protect the message $m_{i}^{j}$ from external leakage, NG performs the following operations: $A_{i}^{j}=\left(\left(m_{i}^{j}⊕r_{i}^{j}\right)\|r_{i}^{j}\right)⊕K_{i}^{j}$, $V_{i}^{j}=H(m_{i}^{j}\|r_{i}^{j}\|ID_{j}\|T_{NG}\left\|K_{i}^{j}\right)$. NG then transmits $M_{1}=\{A_{i}^{j}$, $V_{i}^{j}$, $T_{NG}$, $ID_{j}\}$ to $SM_{j}$ through a public channel.
2. Upon receiving $M_{1}=\{A_{i}^{j}$, $V_{i}^{j}$, $T_{NG}$, $ID_{j}\}$ from NG, $SM_{j}$ checks if the timestamp $T_{NG}$ is within an appropriate range and performs the following operations to verify the message: $\left(m_{i}^{j}⊕r_{i}^{j}\right)\|r_{i}^{j}=A_{i}^{j}⊕K_{i}^{j}$. $SM_{j}$ computes $m_{i}^{j}$ using the extracted $r_{i}^{j}$: $m_{i}^{j}=\left(m_{i}^{j}⊕r_{i}^{j}\right)⊕r_{i}^{j}$. Then, it computes $V_{i}^{j}=H(m_{i}^{j}\|r_{i}^{j}\|ID_{j}\|T_{NG}\left\|K_{i}^{j}\right)$ to verify the integrity of the message. If the verification fails, the protocol is immediately halted. If the verification succeeds, the next phase proceeds.

### 6.3. Second Secure Communication Phase

**Figure 4:**
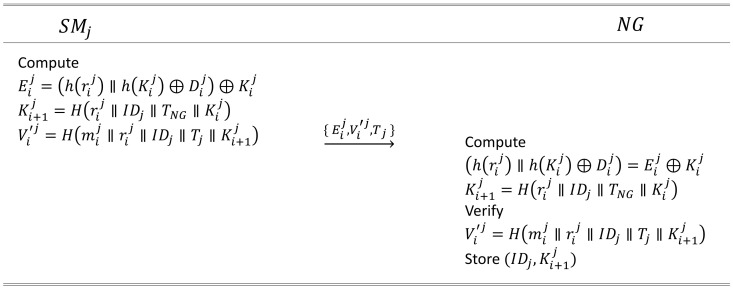
The second authentication phase between smart meter $SM_{j}$ and neighborhood gateway NG proposed in this study.

In this phase, $SM_{j}$ protects and transmits its data report via a public channel to prevent external leakage. NG verifies the data report received from $SM_{j}$ and checks its integrity. The details are presented in Figure 4.

1. To securely send the data report $D_{i}^{j}$ to NG, $SM_{j}$ generates a timestamp $T_{j}$ and performs the following operations: $E_{i}^{j}=\left(h\left(r_{i}^{j}\right)\|h\left(K_{i}^{j}\right)⊕D_{i}^{j}\right)⊕K_{i}^{j}$. It then computes the new key value $K_{i+1}^{j}=H(r_{i}^{j}\|ID_{j}\|T_{NG}\left\|K_{i}^{j}\right)$ and performs the verification $V_{i}^{\prime j}=H(m_{i}^{j}\|r_{i}^{j}\|ID_{j}\|T_{j}\left\|K_{i+1}^{j}\right)$. Then, $SM_{j}$ transmits $M_{2}=\{E_{i}^{j}$, $V_{i}^{\prime j}$, $T_{j}\}$ to NG through a public channel.
2. Upon receiving $M_{2}=\{E_{i}^{j}$, $V_{i}^{\prime j}$, $T_{j}\}$ from $SM_{j}$, NG checks if the timestamp $T_{j}$ is within an appropriate range and performs the following operations for verification $D_{i}^{j}$: $\left(h\left(r_{i}^{j}\right)\|h\left(K_{i}^{j}\right)⊕D_{i}^{j}\right)=E_{i}^{j}⊕K_{i}^{j}$, $D_{i}^{j}=\left(h\left(K_{i}^{j}\right)⊕D_{i}^{j}\right)⊕h\left(K_{i}^{j}\right)$. NG compares $D_{i}^{j}$ with existing reports, and if it matches the established format, it is accepted. When NG computes $K_{i+1}^{j}=H(r_{i}^{j}\|ID_{j}\|T_{NG}\left\|K_{i}^{j}\right)$ and checks the verification $V_{i}^{\prime j}=H(m_{i}^{j}\|r_{i}^{j}\|ID_{j}\|T_{j}\left\|K_{i+1}^{j}\right)$, if the verification is successful, $K_{i+1}^{j}$ replaces the existing $K_{i}^{j}$.

## 7. Security Analysis of the Proposed Scheme

In this section, we describe the formal and informal security analyses of the proposed scheme. The formal security analysis is conducted using ProVerif 2.05 [28], whereas the informal security analysis includes ten different analyses, including providing mutual authentication and resisting replay attacks.

### 7.1. Formal Security Analysis

In this section, we discuss the results of a formal analysis of our scheme conducted using ProVerif. The analysis using ProVerif demonstrates the results of verifying and analyzing the security of the proposed scheme as in several recent studies [29,30,31,32].

We define two types of channels: privateChannel and publicChannel. The reason for setting the publicChannel as private is discussed later when explaining the $SM_{j}$ and NG processes. The constants are set with the $SM_{j}$ID and the NG unique value as *N*. Functions define XOR, concatenate, and two hash operations, and events for $SM_{j}$ and NG are defined for both the first and second authentication phases. The detailed information is provided in Table 2.

The initial and authentication phases of $SM_{j}$ and NG are listed in Table 3 and Table 4. The initial phases of $SM_{j}$ and NG are transmitted through the privateChannel. Subsequently, the first authentication begins. However, the process of omitting the part where *r* is concatenated cannot be implemented using ProVerif. Therefore, to modify it such that NG sends *r* to $SM_{j}$, the publicChannel is set to private to verify the formality.

We verify the results in Table 5 using the queries listed in Table 6. The results are as follows:

- Query inj-event(EVENT) ==> inj-event(EVENT) is true.
- Query not attacker(K) is true.

“Query inj-event(EVENT) ==> inj-event(EVENT) is true” indicates that the event has been verified, and the authentication is successful. This indicates that the event occurred as expected, and under the specified conditions, the authentication mechanism functioned correctly. “Query not attacker(K) is true” indicates that the result of this query is true, which indicates that the attacker could not discover the keys within the array.

### 7.2. Informal Security Analysis

In this section, we present an informal verification of the proposed scheme. Table 7 shows a comparison with previous studies [5,7,10,33]. We conducted ten informal verifications, and the details are as follows.

#### 7.2.1. Provide Mutual Authentication

The proposed scheme verifies the integrity of the message received by $SM_{j}$ from NG during the first authentication phase and the integrity of the message received by NG from $SM_{j}$ during the second authentication phase. Therefore, the proposed scheme provides mutual authentication.

#### 7.2.2. Resist Replay Attack

In the proposed scheme, the decision to proceed with the subsequent operations is based on verifying the timestamps $T_{NG}$ and $T_{j}$ transmitted during the first and second authentication phases, respectively. Therefore, the proposed scheme is resistant to replay attacks.

#### 7.2.3. Resist Smart Meter Impersonation Attack

For an attacker to impersonate $SM_{j}$, they must be able to deceive NG into passing the $V_{i}^{\prime j}$ verification during the second authentication phase. To do this, the attacker must obtain the information necessary to generate $V_{i}^{\prime j}$, which includes $m_{i}^{j}$, $r_{i}^{j}$, and $K_{i+1}^{j}$. The information required to generate $K_{i+1}^{j}$ includes $r_{i}^{j}$ and $K_{i}^{j}$. As the attacker cannot calculate these values from the information $A_{i}^{j}$ and $V_{i}^{j}$ available through the public channel, the attacker cannot impersonate $SM_{j}$.

#### 7.2.4. Resist Extraction of the Secret Key

The only way for an attacker to obtain $K_{i}^{j}$ is by already knowing $m_{i}^{j}$ and $r_{i}^{j}$, and then performing the operation $\left(\left(m_{i}^{j}⊕r_{i}^{j}\right)\|r_{i}^{j}\right)⊕A_{i}^{j}$ or by intercepting it from the private channel. Assuming that interception from the private channel is not possible and because $m_{i}^{j}$ and $r_{i}^{j}$ are neither directly disclosed nor calculated, an attacker cannot obtain $K_{i}^{j}$ in our scheme.

#### 7.2.5. Resist Inferrability of the Message

The message $m_{i}^{j}$ is extracted by performing an XOR operation between $A_{i}^{j}$ and $K_{i}^{j}$. However, as there is no way for an attacker to obtain $K_{i}^{j}$, messages cannot be inferred in our scheme.

#### 7.2.6. Resist Message Altering

In our scheme, message $m_{i}^{j}$ and data report $D_{i}^{j}$ are included in the information contained in $A_{i}^{j}$ and $E_{i}^{j}$, respectively. To verify the integrity of each message $m_{i}^{j}$ and data report $D_{i}^{j}$, ensuring they have not been altered, $V_{i}^{j}$ and $V_{i}^{\prime j}$ are used for verification. Therefore, if an attacker arbitrarily changes the message to create $A_{i}^{j}$ and $E_{i}^{j}$ and attempts to extract the message, it will not pass the verification. Each message and data report can only be verified with the encryption key $K_{i}^{j}$; however, as $K_{i}^{j}$ cannot be extracted by the attacker, the attacker cannot verify the message and data report. Therefore, the proposed scheme resists message alterations.

#### 7.2.7. Resist Injection Attack

In the authentication phases, as message $m_{i}^{j}$ and data report $D_{i}^{j}$ to be transmitted contain the verification variables $V_{i}^{j}$ and $V_{i}^{\prime j}$, it is impossible to perform a data injection attack on the original message and data report. This prevents SQL injections, cross-site scripting, code injections, and other related attacks from becoming feasible.

#### 7.2.8. Provide forward Secrecy

Our scheme employs a method for hashing values that include $K_{i}^{j}$ to generate $K_{i+1}^{j}$. Even if the future key $K_{i+1}^{j}$ is compromised, it is computed as $K_{i+1}^{j}=H(r\|ID_{j}\|T_{NG}\left\|K_{i}^{j}\right)$, which makes it impossible to deduce the value of $K_{i}^{j}$ because of the one-way nature of the hash function. Thus, the proposed scheme provides forward secrecy.

#### 7.2.9. Provide One-Time Pad Key

Our scheme employs a method for hashing values that include $K_{i}^{j}$ to generate the new key $K_{i+1}^{j}$. Thus, the proposed scheme provides a one-time pad key.

#### 7.2.10. Resist Man-in-the-Middle Attack

In the scenario where an attacker accesses the public channel used during the first and second authentication phases of our scheme to carry out a man-in-the-middle attack, the only information they can obtain are $M_{1}=\{A_{i}^{j}$, $V_{i}^{j}$, $T_{NG}$, $ID_{j}\}$ and $M_{2}=\{E_{i}^{j}$, $V_{i}^{\prime j}$, $T_{j}\}$. These values include the smart meter’s identity information and timestamps $T_{NG}$ and $T_{j}$, but among the $A_{i}^{j}=\left(\left(m_{i}^{j}⊕r_{i}^{j}\right)\|r_{i}^{j}\right)⊕K_{i}^{j}$, $V_{i}^{j}=H(m_{i}^{j}\|r_{i}^{j}\|ID_{j}\|T_{NG}\left\|K_{i}^{j}\right)$, $V_{i}^{\prime j}=H(m_{i}^{j}\|r_{i}^{j}\|ID_{j}\|T_{j}\left\|K_{i+1}^{j}\right)$, and $E_{i}^{j}=\left(h\left(r_{i}^{j}\right)\|h\left(K_{i}^{j}\right)⊕D_{i}^{j}\right)⊕K_{i}^{j}$ information, the $V_{i}^{j}$ and $V_{i}^{\prime j}$ values are hashed and therefore unusable. Even if the attacker can see the $A_{i}^{j}$ or $E_{i}^{j}$ values, without knowing the session key, which changes with each session, they cannot recreate these values. Therefore, a man-in-the-middle attack is not feasible.

## 8. Performance Analysis of the Proposed Scheme

In this section, we compare the performance of our paper with related studies. Performance analysis was conducted in the environment of Table 8. The time taken for a hash algorithm was measured as 0.012 ms for symmetric key encryption, decryption was 0.19 ms, and for scalar multiplication in the field, it was 28.03 ms. The computational overhead of the authentication phases for our scheme and related studies [5,7,10,33] is presented in Table 9.

We compute the performance of our scheme in the environment of Table 8 using five hash functions, resulting in a total computational load of 5$T_{h}$ for the neighborhood gateway and 5$T_{h}$ for the smart meter, totaling 10$T_{h}$ = 0.12 ms. According to our findings, Hu et al. [5]’s scheme requires the neighborhood gateway to perform four field multiplications (4$T_{m}$) and use 5$T_{h}$. The smart meter operates at 4$T_{m}$ + 5$T_{h}$, totaling 8$T_{m}$ + 10$T_{h}$ = 224.36 ms. In Garg et al. [33]’s scheme, the neighborhood gateway performs three field multiplications ($T_{m}$), four hash function operations ($T_{h}$), and one symmetric key encryption ($T_{e}$). Additionally, Garg et al.’s smart meter computes at 3$T_{m}$ + 4$T_{h}$ + 1$T_{e}$, totaling 6$T_{m}$ + 8$T_{h}$ + 2$T_{e}$ = 168.656 ms. Similarly, Sureshkumar et al. [7]’s scheme calculates the neighborhood gateway at 3$T_{m}$ + 6$T_{h}$, and the smart meter at 1$T_{m}$ + 4$T_{h}$, totaling 4$T_{m}$ + 10$T_{h}$ = 112.24 ms. Furthermore, we confirmed that the vulnerable scheme by Aghapour et al. [10] involves 4$T_{h}$ for both the neighborhood gateway and the smart meter, resulting in a total of 8$T_{h}$ = 0.096 ms.

## 9. Discussion of Performance

Based on Section 8, we quantify and compare how much better our performance is. The formula we use is as follows:

(6) $$\left(t_{1}-t_{2}\right)/t_{2}$$

According to Formula (6), our scheme demonstrates superior performance by 186,966.67%, 140,546.67%, 93,533.33% and 80.00% compared to Hu et al. [5]’s, Garg et al. [33]’s scheme, Sureshkumar et al. [7]’s scheme and Aghapour et al. [10] scheme. In contrast to other studies [5,7,10,33] which primarily utilize public key or symmetric key cryptography, our scheme mainly uses hash operations to construct lightweight protocols.

According to Table 7, which compares the security aspects of our scheme against others, we found that our scheme performs about 20% worse than Aghapour et al. [10]’s scheme in terms of efficiency. However, our scheme is significantly safer than the proposal by Aghapour et al. [10]. We have developed a scheme that provides a one-time pad key, which Sureshkumar et al. [7]’s scheme failed to do. Moreover, our scheme outperforms the average of the four schemes, including those by Garg et al. [33] and Hu et al. [5], by approximately 105,281.67%.

## 10. Conclusions

In this paper, we proposed a lightweight authentication scheme for SG environments. Our scheme minimizes computational requirements by using only hash functions and XOR operations, and provides security against ten protocol vulnerabilities that previous studies failed to defend, including the extraction of secret keys and the inferrability of the message. We demonstrate that our scheme satisfies the security requirements using ProVerif, a formal verification tool. Moreover, in terms of performance, our scheme shows a superior computational speed of 105,281.67% compared with other schemes.

## Figures and Tables

**Table 1:** Notations used in this paper.

Notations	Description
$SM_{j}$	*j*-th smart meter
NG	Neighborhood gateway
$ID_{j}$	$SM_{j}$’s identification
$m_{i}^{j}$	*i*-th message for $SM_{j}$
$D_{i}^{j}$	Data report of *i*-th $SM_{j}$
$V_{i}^{j}$, $V_{i}^{\prime j}$	Verification
$K_{i}^{j}$	*i*-th secret key for $SM_{j}$
$r_{i}^{j}$	*i*-th random number for $SM_{j}$
*h* (·), *H* (·)	One-way hash function
X‖Y	Concatenation operator
⊕	Bitwise XOR operator
$T_{NG}$, $T_{j}$	Timestamp for NG and $SM_{j}$

**Table 2:** ProVerif code for defining values and functions.

(*—-channels—-*)
free privateChannel:channel [private].
free publicChannel:channel [private].

(*—-constants—-*)
free ID:bitstring [private].
free N:bitstring [private].

(*—-shared key—-*)
free K:bitstring [private].

(*—-functions—-*)
fun xor(bitstring, bitstring):bitstring.
fun concat(bitstring, bitstring):bitstring.
fun h(bitstring):bitstring.
fun H(bitstring):bitstring.
equation forall a:bitstring, b:bitstring; xor(xor(a, b), b) = a.

(*—-events—-*)
event startfstS(bitstring).
event endfstS(bitstring).
event startfstN(bitstring).
event endfstN(bitstring).
event start2ndS(bitstring).
event end2ndS(bitstring).
event start2ndN(bitstring).
event end2ndN(bitstring).

**Table 3:** ProVerif code for the SM.

(*—-SMj process—-*)
let SMj =
out(privateChannel, (ID));
in(privateChannel, (XK:bitstring));
event startfstS(ID);
in(publicChannel, (XA:bitstring, XV:bitstring, XT:bitstring, XXID:bitstring, Xr:bitstring));
let P = xor(xor(XA, XK), XA) in
let Xm = xor(P, Xr) in
let XXV = H(concat(concat(Xm, Xr), concat(concat(XXID, XT), XK))) in
event endfstS(ID);
event start2ndS(ID);
if XV = XXV then
new Tj:bitstring;
new D:bitstring;
let E = xor(xor(concat(h(Xr), h(XK)), D), XK) in
let newK = H(concat(concat(Xr, XXID), concat(XT, XK))) in
let Vp = H(concat(concat(Xm, Xr), concat(concat(XXID, Tj), newK))) in
out(publicChannel,(E, Vp, Tj));
event end2ndS(ID).

**Table 4:** ProVerif code for the neighborhood gateway.

(*—-NG process—-*)
let NG =
in(privateChannel, (XID:bitstring));
out(privateChannel, (K));
event startfstN(N);
new r:bitstring;
new m:bitstring;
new T:bitstring;
let A = xor(xor(m, r), K) in
let V = H(concat(concat(m, r), concat(concat(XID, T), K))) in
out(publicChannel,(A, V, T, XID, r));
event endfstN(N);
event start2ndN(N);
in(publicChannel,(XE:bitstring, XVp:bitstring, XTj:bitstring));
let PP = xor(XE, K) in
let XD = xor(PP, concat(h(r), h(K))) in
let XnewK = H(concat(concat(r, XID), concat(T, K))) in
let XXVp = H(concat(concat(m, r), concat(concat(XID, XTj), XnewK))) in
if XVp = XXVp then
event end2ndN(N).

**Table 5:** ProVerif query results.

Query inj-event(endfstS(IDj)) ==> inj-event(startfstS(IDj)) is true.
Query inj-event(end2ndS(IDj)) ==> inj-event(start2ndS(IDj)) is true.
Query inj-event(endfstN(IDj)) ==> inj-event(startfstN(IDj)) is true.
Query inj-event(end2ndN(IDj)) ==> inj-event(start2ndN(IDj)) is true.
Query not attacker(K[]) is true.

**Table 6:** ProVerif code for queries.

(*—-queries—-*)
query IDj:bitstring; inj-event(endfstS(IDj)) ==> inj-event(startfstS(IDj)).
query IDj:bitstring; inj-event(end2ndS(IDj)) ==> inj-event(start2ndS(IDj)).
query IDj:bitstring; inj-event(endfstN(IDj)) ==> inj-event(startfstN(IDj)).
query IDj:bitstring; inj-event(end2ndN(IDj)) ==> inj-event(start2ndN(IDj)).
query attacker(K).

(*—-process—-*)
process
((!SMj)|(!NG))

**Table 7:** Comparison of security features.

Security Features	Sureshkumar et al. [7]	Garg et al. [33]	Hu et al. [5]	Aghapour et al. [10]	Ours
Provide Mutual Authentication	O	O	O	O	O
Resist Replay Attack	O	O	O	O	O
Resist Smart Meter Impersonation Attack	O	O	O	O	O
Resist Extraction of the Secret Key	O	O	O	O	O
Resist Inferrability of the Message	O	O	O	X	O
Resist Message Altering	O	O	O	X	O
Resist Injection Attack	O	O	O	O	O
Provide Forward Secrecy	O	O	O	O	O
Provide One-time Pad Key	X	O	O	O	O
Resist Man-in-the-Middle Attack	O	O	O	X	O

**Table 8:** Development environment.

Item	Value
CPU	Intel(R) Core(TM) i7-8565U CPU @ 1.80 GHz 1.99 GHz (Intel, Santa Clara, CA, USA)
RAM	16.0 GB
OS	Windows 10 Home
Software	JDK 17
Security level	secp521r1 ECC

**Table 9:** Comparisons of computational costs (ms).

Schemes	Hu et al. [5]	Garg et al. [33]	Sureshkumar et al. [7]	Aghapour et al. [10]	Ours
NG, SP	$4T_{m}+5T_{h}$	$3T_{m}+4T_{h}+1T_{e}$	$3T_{m}+6T_{h}$	4$T_{h}$	5$T_{h}$
	=112.18	=84.328	=84.162	=0.048	=0.06
Smart Meter(SM)	$4T_{m}+5T_{h}$	$3T_{m}+4T_{h}+1T_{e}$	$1T_{m}+4T_{h}$	4$T_{h}$	$5T_{h}$
	=112.18	=84.328	=28.078	=0.048	=0.06
Total	$8T_{m}+10T_{h}$	$6T_{m}+8T_{h}+2T_{e}$	$4T_{m}+10T_{h}$	$8T_{h}$	$10T_{h}$
	=224.36	=168.656	=112.24	=0.096	=0.12

## Data Availability

Data are contained within the article.
